# Radiological, functional, and anatomical outcome in patients with osteoarthritic knee undergoing high tibial osteotomy

**DOI:** 10.1051/sicotj/2019009

**Published:** 2019-05-03

**Authors:** Muhammad Khurram Habib, Zeeshan Ali Khan

**Affiliations:** 1 DHQ Hospital Faisalabad Pakistan

**Keywords:** Osteoarthritis knee, High tibial osteotomy, Functional outcome

## Abstract

*Objective*: To determine the radiological, functional, and anatomical outcome in patients with osteoarthritic knee undergoing high tibial osteotomy (HTO).

*Design of study*: Descriptive case series.

*Study duration and settings*: The present study was a descriptive case series carried out at the Orthopedic Departments of District Head Quarter Hospital Faisalabad affiliated with Faisalabad Medical University, Faisalabad from Jan 2014 to March 2018.

*Methodology*: This study involved 40 patients of both genders, aged between 40 and 65 years having advanced degenerative disease of knee limited to medial compartment of joint. These patients were treated by medial open wedge high tibial osteotomy (OWHTO) and outcome was evaluated after 5 years of surgery in terms of radiological knee mechanics, functional outcome scores, and arthroscopic evidence of cartilage regeneration in the medial compartment. A signed written consent was taken from every patient.

*Findings:* There was a female predominance with a male-to-female ratio of 1:4. The mean age of the patients was 53.2 ± 6.9 years. The values of the radiographic parameters significantly changed from pre-operative condition after HTO; mechanical tibiofemoral angle [MTFA, (−8.1 ± 1.2° vs. 2.5 ± 1.2°; *p*-value < 0.0001)], tibial plateau inclination [TPI, (5.3 ± 1.1° vs. 3.4 ± 1.1°; *p*-value < 0.0001)], knee joint line orientation relative to the ground [G-KJLO, (0.3 ± 0.1° vs. 4.6 ± 1.5°; *p*-value < 0.0001)], and ankle joint line orientation relative to the ground [G-AJLO (8.3 ± 3.2° vs. 2.3 ± 1.7°; *p*-value < 0.0001)]. There was significant improvement in patient’s functional status; KOOS-ADL score (45.5 ± 7.8 vs. 73.7 ± 8.6; *p*-value < 0.0001), International Knee Documentation Committee (IKDC) score (42.4 ± 6.9 vs. 68.5 ± 12.7; *p*-value < 0.0001), International Knee Society (IKS) score (149.4 ± 11.9 vs. 179.4 ± 10.2; *p*-value < 0.0001), Knee Society Score [KSS, (54.2 ± 5.6 vs. 69.7 ± 12.7; *p*-value < 0.0001)], and Hospital for Special Surgery [HSS, (50.8 ± 3.3 vs. 64.8 ± 10.7; *p*-value < 0.0001)]. 42.5% patients showed excellent regeneration of femoral and 30.0% patients showed excellent regeneration of tibial cartilage in the medial compartment.

*Conclusion*: By significantly alternating the knee biomechanics, HTO was found to unload the medial compartment leading to regeneration of the articular cartilage and significant improvement in patient’s symptoms and quality of life. It is therefore recommended in the management of patients with arthritic changes limited to medial compartment only.

## Introduction

Osteoarthritis (OA), is a highly prevailing joint disease, affecting about 18–20% of women and 8–10% of men over 60 years of age [1, 2]. Although osteoarthritis may affect any joint, the hip and knee joints are more frequently involved [1]. OA affects the entire joint including synovium, subchondral bone, and articular cartilage and leads to joint stiffness, pain, crepitus, reduced range of movement, limited function and varying degrees of local inflammation [3, 4]. Osteoarthritis is a chronic condition and currently there is no cure for it [1]. The management of knee osteoarthritis can be grouped into first, second, and third lines of treatment [5–7]. The first line of treatment comprises patient’s education, physiotherapy, and weight reduction [7], while the second line of treatment consists of drug therapy for the control of pain, assistive devices for activities of daily living, along with the first line of treatment [7]. The third line of treatment comprises surgery where total knee arthroplasty (TKA) is the treatment of choice for end-stage arthritic joint [5–7].

Debeyre and Patte in 1951 [8] first described the opening wedge high tibial osteotomy (OWHTO). Though initially proposed as a definitive management approach for patients with isolated medial compartment knee osteoarthritis, the dawn of the total knee replacement (TKR) led to the high tibial osteotomy (HTO) falling out of favor particularly in the lower demand and older patient population [8, 9]. Knee osteoarthritis can affect younger, more dynamic patients due to trauma, sporting injuries, work, and overuse. TKA is frequently not a feasible option in such patients due to amplified levels of cyclical loading of the knee, leading to more rapid wear and the problems of premature implant failure [10]. Patient’s request for a surgical resolution of pain while preserving the vigorous lifestyle continues and OWHTO gives an opportunity to buy some time and delay TKR [8, 9].

Data on long term outcome of OWHTO are scarce and only few studies have reported outcome after 5 years of surgery [8, 9]. Also, there was no such local published material which necessitated the present study to determine the clinical, radiological, and anatomical outcome of OWHTO in patients with medial compartment osteoarthritis of knee.

## Material and methods

The present study was a descriptive case series carried out at the Orthopedic Departments of District Head Quarter Hospital Faisalabad affiliated with Faisalabad Medical University, Faisalabad from Jan 2014 to March 2018. Sample size of 40 cases was calculated with 95% confidence interval and 80% power of test considering expected mean International Knee Society (IKS) score function score to be 84 ± 15.5 before and 95 ± 8.9 after HTO in patients with medial compartment OA knee [11]. Patients of both genders, aged between 40 and 65 years having advanced degenerative disease of knee (grade 3 or 4 on radiological assessment according to Kellgren and Lawrence) limited to medial compartment of joint, varus angulation of more than 4°, and arthroscopic full-thickness articular cartilage deficiencies in the medial compartment were included in this study. Patients ≥ 65 years of age, with secondary arthritis (after trauma, infection, and ligamentous instability of joint), advanced patellofemoral arthritis, ROM < 90°, flexion contracture ≥15°, joint instability and lateral tibial thrust ≥1 cm, and rheumatoid arthritis were not included in the study.

**Figure 1:**
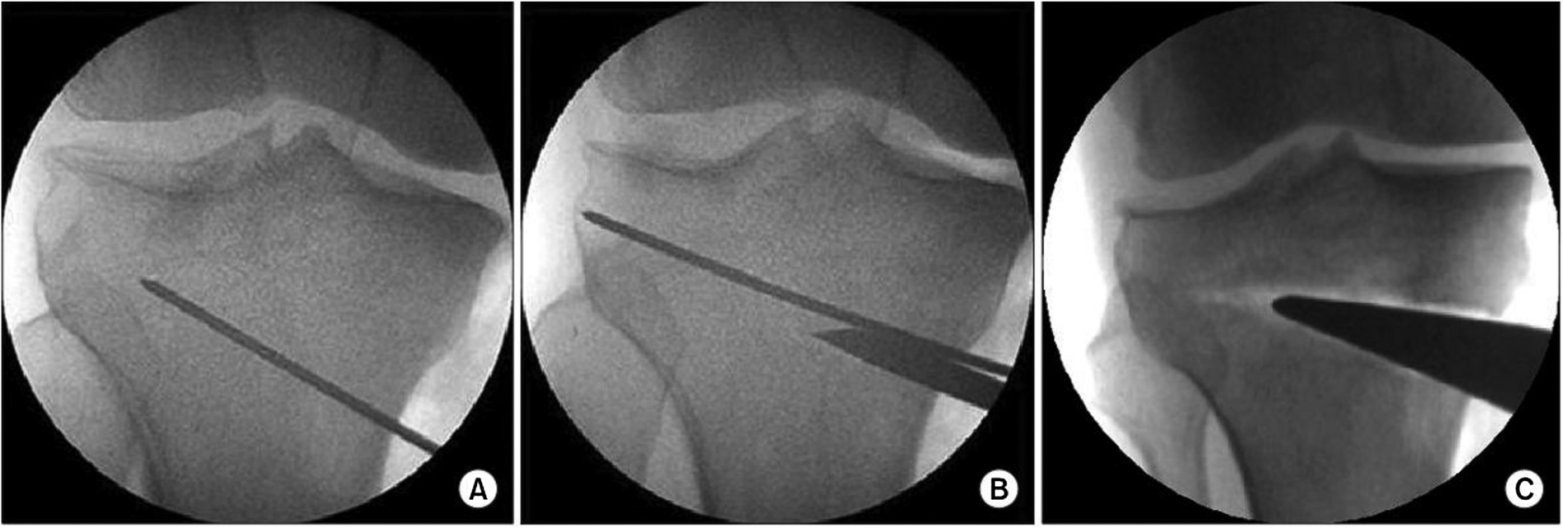
OWHTO. (A) Guide wire is placed, (B) osteotomy is done with a saw, (C) the medial tibia is released with a wedge.

The procedure was performed through a standardized technique by the senior surgeon under tourniquet control. The patient was placed supine on a radiolucent operating table. A 5-cm longitudinal incision was placed over the center between the tibial tuberosity and the posteromedial aspect of the tibia underneath the joint line. Once the medial border of the patellar tendon was identified, subperiosteal dissection was done from the tibial tuberosity to the posteromedial aspect of the tibia. Two guide wires were placed 3.5–4 cm below the medial joint line passed diagonally 1 cm underneath the lateral margin of the tibia (Figure 1A). The appropriate location was confirmed with fluoroscope. A tibial osteotomy was then completed directly underneath the guide wires with the help of a saw (Figure 1B). Afterward, a regulated wedge was introduced till the osteotomy was opened to the preferred extent (Figure 1C). Once the anticipated degree of correction was attained, internal fixation was performed with a metal plate.

With partial weight bearing, early joint exercises allowing 0°–90° of motion were started with the knee secured in a hinged brace for 6–8 weeks. Over 6–12 weeks, gradually weight bearing was increased, while the knee brace was cast-off. Spreading over regular intervals, *X*-rays were used to evaluate bone union and maintenance of correction. Outcome was assessed radiologically; by change in mechanical tibiofemoral angle (MTFA), tibial plateau inclination (TPI), knee joint line orientation relative to the ground (G-KJLO), and ankle joint line orientation relative to the ground (G-AJLO) angles, functionally; by improvement in Knee-Injury and Osteoarthritis Outcome Score (KOOS), International Knee Documentation Committee (IKDC) score, IKS, Hospital for Special Surgery (HSS) knee scores, and Knee Society Score (KSS) and anatomically by arthroscopic evidence of regeneration of articular cartilage in the medial compartment graded as excellent (100%), good (≥50%), and poor (<50%) after 5 years of surgery.

All the data were entered and analyzed through Statistical Package for the Social Sciences (SPSS). Paired sample *t*-test was used to compare mean change in numerical variables before and after treatment.

## Results

The age of the patients ranged from 40 years to 65 years with a mean of 53.2 ± 6.9 years. There were 8 (20.0%) male and 32 (80.0%) female patients in the study group with a male-to-female ratio of 1:4.

**Figure 2:**
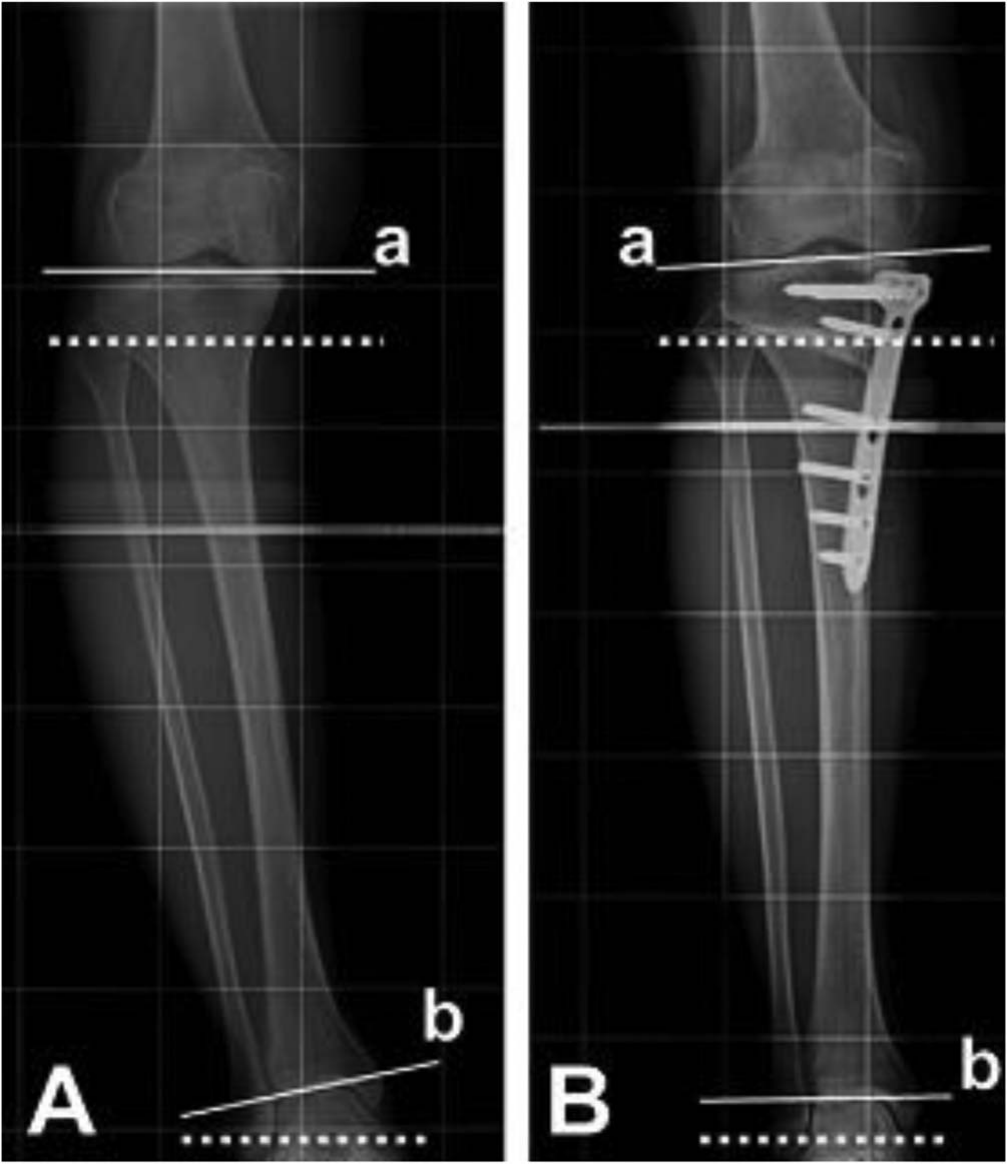
*X*-ray describing changes of the KJLO and the AJLO comparative to the ground.

**Figure 3:**
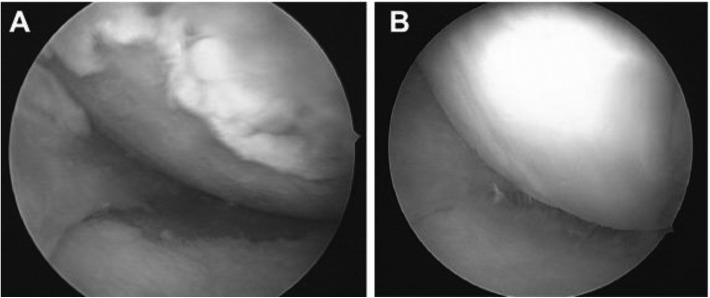
Medial compartment articular cartilage. (a) Before surgery and (b) after surgery.

The values of the radiographic parameters significantly changed from pre-operative condition after HTO. Remarkably after HTO, the G-KJLO increased much less than did the TPA, owing to a reciprocal change of the AJLO relative to the ground (Table 1, Figure 2). We found that the decrease of the AJLO relative to the ground backed the relative small change of the knee joint line orientation; the sum of changes in those two alignments came close to the overall change of the MTFA after HTO. Various scores assessing functional outcome improved significantly than pre-operative values as shown in Table 1. Arthroscopic evaluation revealed marked improvement in femoral and tibial articular cartilage in the medial compartment owing to the unloading effect of osteotomy and regeneration of fibrocartilage (Table 2, Figure 3).

**Table 1:** Comparison of various outcome measures before and after osteotomy.

Outcome	Pre-operative	Post-operative	*p*-value
Radiological outcome			
MTFA (°)	−8.1 ± 1.2	2.5 ± 1.2	<0.0001*
TPI (°)	5.3 ± 1.1	3.4 ± 1.1	<0.0001*
G-KJLO (°)	0.3 ± 0.1	4.6 ± 1.5	<0.0001*
G-AJLO (°)	8.3 ± 3.2	2.3 ± 1.7	<0.0001*
Functional outcome			
KOOS-ADL score	45.5 ± 7.8	73.7 ± 8.6	<0.0001*
IKDC score	42.4 ± 6.9	68.5 ± 12.7	<0.0001*
IKS score	149.4 ± 11.9	179.4 ± 10.2	<0.0001*
KSS	54.2 ± 5.6	69.7 ± 12.7	<0.0001*
HSS	50.8 ± 3.3	64.8 ± 10.7	<0.0001*

Paired sample *t*-test.

* Observed difference was statistically significant.

**Table 2:** Regeneration of articular cartilage.

Characteristics	Femoral	Tibial
Excellent	17 (42.5%)	12 (30.0%)
Good	18 (45.0%)	20 (50.0%)
Poor	5 (12.5%)	8 (20.0%)

## Discussion

HTO can delay the need for TKA and is advised for the treatment of osteoarthritis of knee joint in young and active patients for relief of pain while preserving normal activity levels [12]. Though the use of HTO has dropped lately due to the development of TKA, it is undeniable that proper patient choice, accurate surgical preparation, and appropriate surgical technique can provide promising outcomes of HTO [13, 14]. The purpose of the present study was to evaluate the radiological, functional, and anatomical outcome of HTO in carefully selected cohort of patients with medial compartment OA knee.

Our findings are consistent with already published literature where Lee et al. [15] reported similar changes in the biomechanics of knee after medial open wedge high tibial osteotomy (OWHTO); MTFA (−8.3 ± 2.3° vs. 2.5 ± 2.2°; *p*-value < 0.0001), TPI (5.6 ± 2.1° vs. 3.4 ± 3.0°; *p*-value < 0.0001), G-KJLO (0.3 ± 2.1° vs. 4.4 ± 2.6°; *p*-value < 0.0001), and G-AJLO (8.8 ± 3.2° vs. 2.0 ± 3.9°; *p*-value < 0.0001). In another similar study Schuster et al. [16] reported that International Knee Documentation Committee score improved significantly after HTO (44 ± 11 pre-operatively to 70 ± 13 post-operatively; *p*-value < 0.001) in line with the present study. Schuster et al. [16] also reported similar frequency of excellent cartilage regeneration on femoral (40.9%) and tibial (29.4%) condyles in the medial compartment of the joint. Polat et al. [17] in a similar study reported similar significant improvement in KSS (56.3 ± 13.4 vs. 70.3 ± 14.9; *p*-value ≤ 0.05) and the HSS score (51.3 ± 9.7 vs. 64.7 ± 13.5; *p*-value ≤ 0.05). Similar improvement in KOOS-activities of daily living score (48.3 ± 20.1 vs. 78.5 ± 19.8; *p*-value ≤ 0.05) and IKS score (154 ± 26.1 vs. 185 ± 12.1; *p*-value ≤ 0.05) has been reported by Roos et al. [18] and Brosset et al. [11], respectively.

The present study is first of its kind in local population and adds to the already published international research on the topic. The strengths of the present study are longer follow-up of 5 years and arthroscopic, radiological, and functional evaluation of patient’s outcome. In the light of this evidence, it can be advocated that patients with OA knee who are particularly young with changes limited to medial compartment of the joint should be offered HTO to improve the patient condition while buying some time before the patient needs TKR.

However, some patients benefitted initially by a HTO may later require a knee replacement, considering the severity of arthritis, patient’s pain, and difficulty in performing activities of daily life [19, 20]. In these patients, knee replacement becomes difficult due to loss of bone stock, modified joint line, subsequent patella infera, and is particularly challenging in those with more severe overcorrection [19]. However, in skillful hands that may not adversely affect the outcome of TKR as shown by recent studies comparing primary TKR with versus without prior HTO which did not reveal any significant waning of outcome [20–22], thus further encouraging the use of HTO particularly in young patients with unicompartmental disease to buy more time before joint replacement is unavoidable.

A very strong limitation to the present study was that we only performed medial open wedge osteotomy and did not compare the outcome between medial open wedge and lateral closing wedge osteotomy which would have further helped in the selection of appropriate approach and implant. Such a study is highly recommended in future research.

## Conclusion

By significantly alternating the knee biomechanics, HTO was found to unload the medial compartment leading to regeneration of the articular cartilage and significant improvement in patient’s symptoms and quality of life. It is therefore recommended in the management of patients with arthritic changes limited to medial compartment only.
